# Supplementary material for: Ectoderm to mesoderm transition by down-regulation of actomyosin contractility
Source: PLoS Biol. 2021 Jan 6;19(1):e3001060. doi: 10.1371/journal.pbio.3001060 (PMC7815211; doi:10.1371/journal.pbio.3001060)
Supplement: S1 Table — (RTF) [file pbio.3001060.s001.rtf]

S1 Table
List of mRNA used in this study with injected amounts
All plasmids are based on the pCS2+MTYFP vector [34].
Plasmid 


	mRNA injected per blastomere at 2 cell stage (pg)	
mCherry (membrane-targeted YFP)	50-250	
mYFP (membrane-targeted YFP)	50-250	
C-cadherin-dTomato	1000	
Vinculin-Cherry	125-250	
Paxillin-YFP	250	
Myosin light chain (MLC)-Cherry	500	
Non-muscle myosin heavy chain 2A (NMHC2A)-YFP	1000	
Non-muscle myosin heavy chain 2B (NMHC2B)-YFP	1000	
Rnd1-YFP	125-500	
Shirin-YFP	75-300	
ShirinR488A-YFP	75	
