# Supplementary material for: Ectoderm to mesoderm transition by down-regulation of actomyosin contractility
Source: PLoS Biol. 2021 Jan 6;19(1):e3001060. doi: 10.1371/journal.pbio.3001060 (PMC7815211; doi:10.1371/journal.pbio.3001060)
Supplement: S2 Table — (PDF) [file pbio.3001060.s002.pdf]

S2 Table.

List of morpholinos with injected amounts

Target	Sequence	Amount/injected blastomore
C-cadherin	CCACCGTCCCGAACGAAGCCTCAT	40ng
Rnd1a<br>Rnd1b	AGTACGGTGGGACAAATCCAACAAC<br>ACAAGTCCTAATTAAAAGCTCCACG	20ng+<br>20ng
ShirinS2a<br>ShirinS2b	CTGGCCTCCCATTTTCCCAGAAGGT<br>GCCTCCCATTTTCCCAGAGACACGA	20ng+<br>20ng
