# Supplementary material for: Ectoderm to mesoderm transition by down-regulation of actomyosin contractility
Source: PLoS Biol. 2021 Jan 6;19(1):e3001060. doi: 10.1371/journal.pbio.3001060 (PMC7815211; doi:10.1371/journal.pbio.3001060)
Supplement: S1 Fig — (Related to Fig 1) (A) Mode of mesoderm locomotion. Consecutive frames from time lapse of mYFP labeled mesoderm cells migrating on FN. The behavior of the central cell is highlighted: The cell emits 1 or multiple protrusions (red arrows). One of the protrusions becomes a tail (yellow arrowhead) as the cell stretches toward another direction, and eventually retracts (red arrowheads). (B) Quantification of accumulation of Vinculin-Cherry in FAs: linearity between fluorescence levels in FA and total intensity (related to Fig 1A–1D). Because Vinculin-Cherry expression levels vary from cell to cell, quantification was performed for individual cells by measuring fluorescence in bright clusters (corresponding to FAs) and in the total ventral cell surface (pink on the diagram). The plot shows the average intensity of the ventral surface versus the average intensity in FAs for control mesoderm cells in 1 experiment, each dot corresponding to a single cell. It shows that accumulation at FAs is proportional to total expression levels over a wide range. Refer to S1 Data. Linearity was similarly verified for each experiment. FA, focal adhesion; FN, fibronectin; mYFP, membrane-targeted yellow fluorescent protein. (PDF) [file pbio.3001060.s003.pdf]

S1 Fig

A

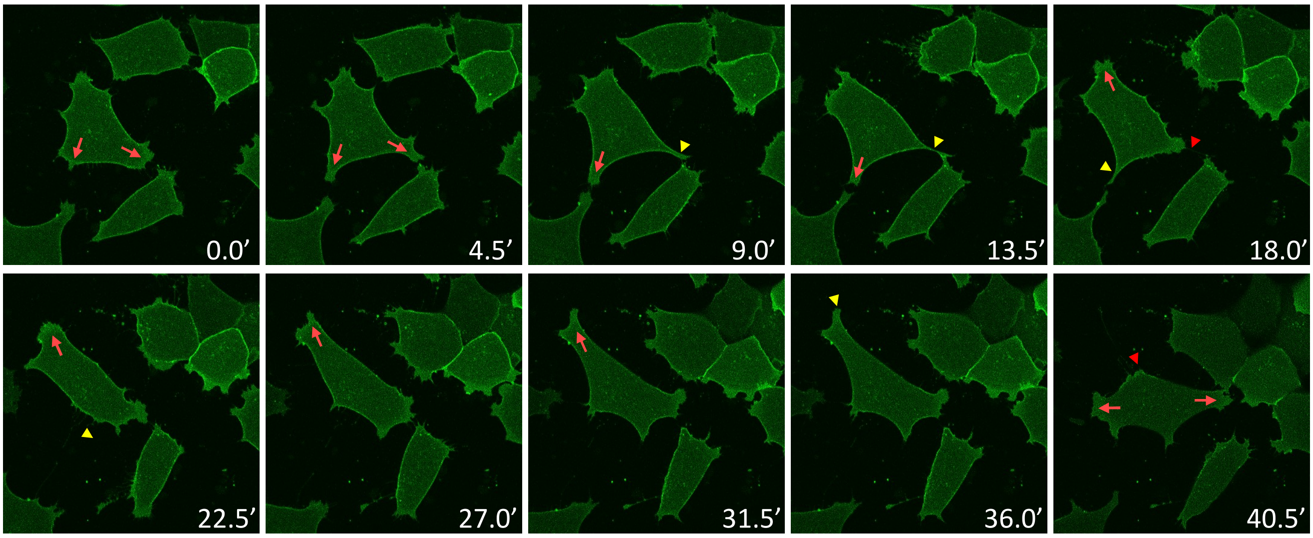

A) Mode of mesoderm locomotion (Related to Figure 1) Consecutive frames from time lapse of mYFP labelled mesoderm cells migrating on FN. The behaviour of the central cell is highlighted: The cell emits one or multiple protrusions (red arrows). One of the protrusions becomes a tail (yellow arrowhead) as the cell stretches toward another direction, and eventually retracts (red arrowheads).

B

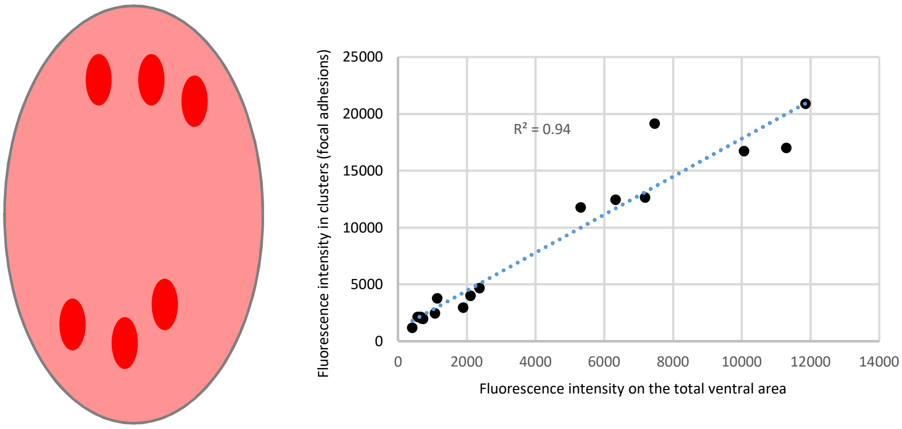

B) Quantification of accumulation of Vinculin-Cherry in focal adhesions: Linearity between fluorescence levels in focal adhesion and total intensity (Related to Figure 1A-D) Because Vinculin-Cherry expression levels vary from cell to cell, quantification was performed for individual cells by measuring fluorescence in bright clusters (corresponding to focal adhesions) and in the total ventral cell surface (pink on the diagram). The plot shows the average intensity of the ventral surface versus the average intensity in focal adhesions for control mesoderm cells in one experiment, each dot corresponding to a single cell. It shows that accumulation at focal adhesions is proportional to total expression levels over a wide range. Linearity was similarly verified for each experiment.
