# Supplementary material for: Ectoderm to mesoderm transition by down-regulation of actomyosin contractility
Source: PLoS Biol. 2021 Jan 6;19(1):e3001060. doi: 10.1371/journal.pbio.3001060 (PMC7815211; doi:10.1371/journal.pbio.3001060)
Supplement: S2 Fig — (A–C) Differential MLC accumulation at the cell cortex. Ectoderm and mesoderm cells expressing MLC-Cherry (MLC-Che) and mYFP. (A) Ectoderm cells show strong accumulation around the cell body (arrows) and part of the blebs (arrowhead). (B) Mesoderm cells show irregular cortical MLC, mostly at the concave regions near or between protrusion. (C) Quantification of cortical MLC, expressed as the ratio of cortical /cytoplasmic fluorescence intensities. Blebs and protrusions were excluded from the measurements. Statistical comparison using 2-sided Student t test. Refer to S1 Data. Scale bars: A’ 5 μm, B’ 10 μm, B” 5 μm. (D–K) Subcellular localization of Rock1-YFP and Rock2-YFP in ectoderm and mesoderm cells. Selected single planes from live confocal microscopy, either near the glass (ventral) or about 5–10 μm above (medial). Concave white arrowheads point at examples of Rock1/2 accumulation. (D, E, H, I) Localization relative to the cell cortex and to Vinculin-Cherry labeled cell-matrix adhesive structures (red arrowheads). (F, G, J, K) Localization relative to cell–cell contacts, marked by cadherin-dTomato (red arrows). (D, E) In the ectoderm, Rock1 and 2 have both a cortical localization. Levels are low on the ventral side inside the adhesive ring, but stronger outside of the ring, particularly for Rock2. (F, G) Levels are very low at cell–cell contacts. (H, I) In the ventral face of mesoderm cells, Rock1 tends to be enriched in the central part, Rock2 at the periphery of the protrusions. Both are low at FAs. They both accumulate at the cortex along cell free edges (medial planes). (J, K) Levels are low at cell–cell contacts. y: autofluorescence of yolk platelets, abundant in mesoderm cells. FA, focal adhesion; MLC, myosin light chain; mYFP, membrane-targeted YFP; Rock, Rho-kinases. (PDF) [file pbio.3001060.s004.pdf]

S2 Fig

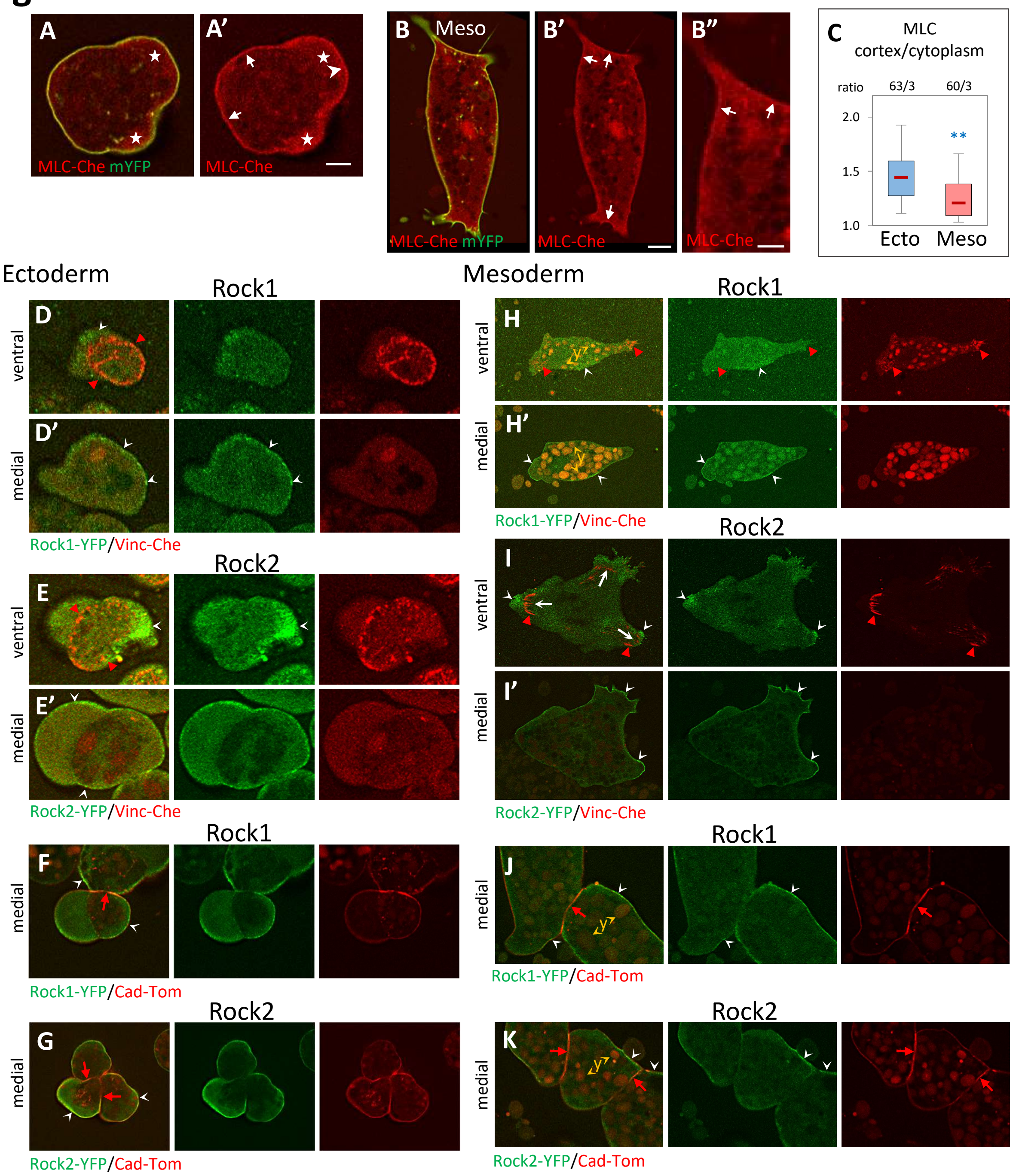

Localization of MLC and Rock (Related to Figure 2)

A-C) Differential MLC accumulation at the cell cortex. Ectoderm and mesoderm cells expressing MLC-Cherry (MLC-Che) and mYFP. A) Ectoderm cells show strong accumulation around the cell body (arrows) and part of the blebs (arrowhead). B) Mesoderm cells show irregular cortical MLC, mostly at the concave regions near or between protrusion. C) Quantification of cortical MLC, expressed as the ratio of cortical /cytoplasmic fluorescence intensities. Blebs and protrusions were excluded from the measurements. Statistical comparison using two-sided Student's *t-test*. Scale bars: A' 5µm, B' 10µm, B" 5µm.

D-K) Subcellular localization of Rock1-YFP and Rock2-YFP in ectoderm and mesoderm cells. Selected single planes from live confocal microscopy, either near the glass (ventral), or about 5-10µm above (medial). Concave white arrowheads point at examples of Rock1/2 accumulation. D,E,H,I) Localization relative to the cell cortex and to vinculin-Cherry labelled cell-matrix adhesive structures (red arrowheads). F,G,J,K) Localization relative to cell-cell contacts, marked by cadherin-dTomato (red arrows). D,E) In the ectoderm, Rock1 and 2 have both a cortical localization. Levels are low on the ventral side inside the adhesive ring, but stronger outside of the ring, particularly for Rock2. F,G) Levels are very low at cell-cell contacts. H,I) In the ventral face of mesoderm cells, Rock1 tend to be enriched in the central part, Rock2 at the periphery of the protrusions. Both are low at FAs. They both accumulate at the cortex along cell free edges (medial planes). J,K) Levels are low at cell-cell contacts. Y: autofluorescence of yolk platelets, abundant in mesoderm cells.
