# Supplementary material for: Ectoderm to mesoderm transition by down-regulation of actomyosin contractility
Source: PLoS Biol. 2021 Jan 6;19(1):e3001060. doi: 10.1371/journal.pbio.3001060 (PMC7815211; doi:10.1371/journal.pbio.3001060)
Supplement: S3 Fig — (Related to Fig 2) (A, B) Area expansion for single cells after treatment with Rock inhibitors Y27632 (50 μM) and H1125 (1 μM). Average and SD of 107 cells (A) and 34 cells (B). (C) Changes in vinculin distribution. Images from a time-lapse movie of a small group of 3 cells expressing Vinculin-Cherry, treated at time = 0 with Y27632. Filled arrowheads: ring-like adhesion; concave arrowheads: FAs. Scale bars: 10 μm. (D, E) Opposite effects of Rock and MLCK inhibition on cell adhesion. Ectoderm and mesoderm adhesion to FN or cadherin was measured after treatment with Rock inhibitors Y27632 (Y, 50 μM), H1125 (H, 1 μM), or the MLCK inhibitor ML7. Five experiments, a total of 1,000–2,000 cells/conditions. Statistical comparison to control ectoderm or mesoderm, comparing the % adherent cells/experiment, pairwise 2-sided Student t test. Refer to S1 Data. FA, focal adhesion; FN, fibronectin; SD, standard deviation. (PDF) [file pbio.3001060.s005.pdf]

S3 Fig

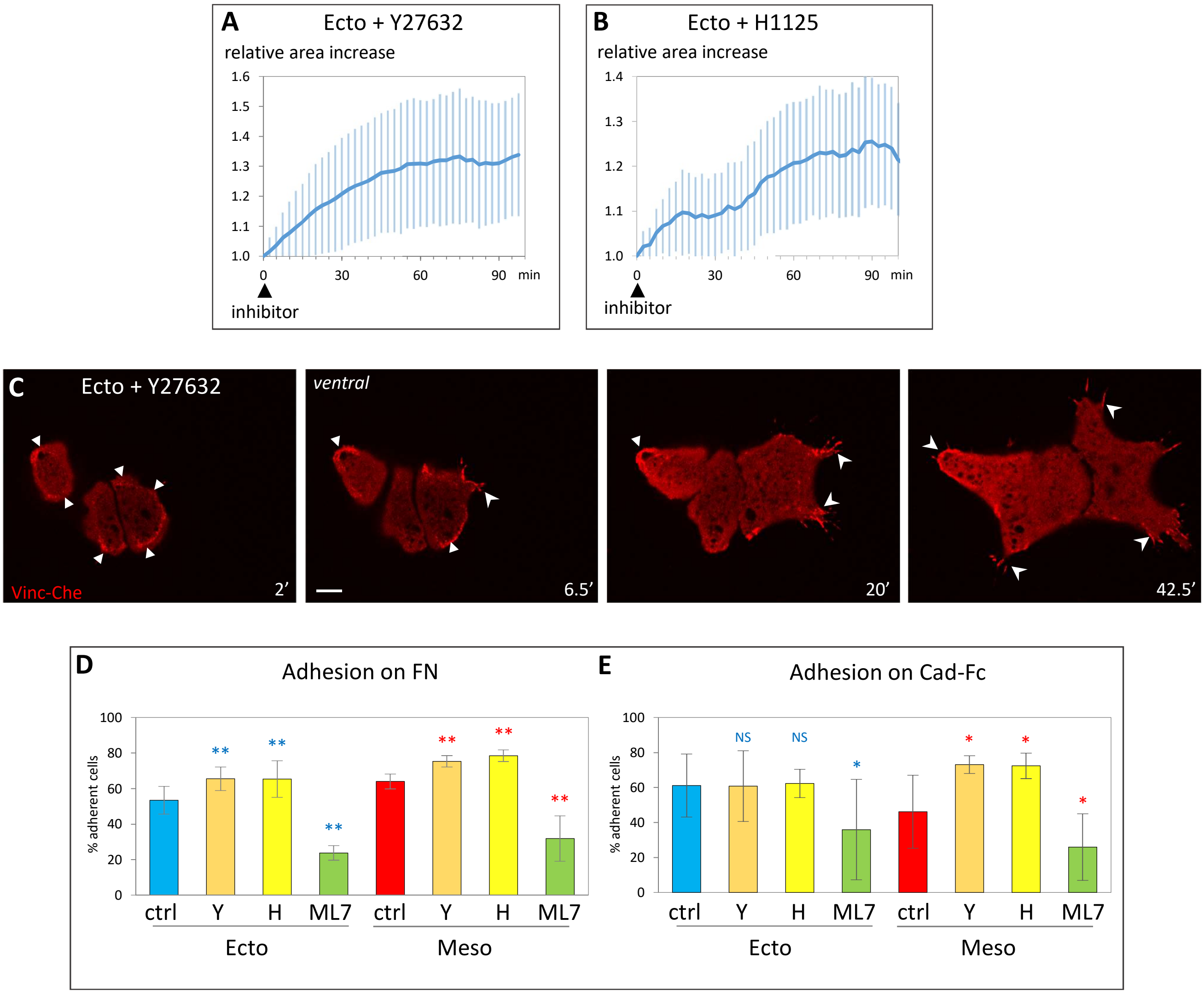

Related to Figure 2: **A,B) Area expansion** for single cells after treatment with Rock inhibitors Y27632 (50µM) and H1125 (1µM)**.** Average and SD of 107 cells (A) and 34 cells (B). **C) Changes in vinculin distribution.** Images from a time lapse movie of a small group of three cells expressing Vinculin-Cherry, treated at time = 0 with Y27632. Filled arrowheads: ring-like adhesion; Concave arrowheads: FAs. Scale bars: 10µm. **D,E) Opposite effects of Rock and MLCK inhibition on cell adhesion.** Ectoderm and mesoderm adhesion to FN or cadherin was measured after treatment with Rock inhibitors Y27632 (Y, 50µM), H1125 (H, 1µM), or the MLCK inhibitor ML7. 5 experiments, total 1000-2000 cells/conditions. Statistical comparison to control ectoderm or mesoderm, comparing the % adherent cells/experiment, pairwise two-sided Student's *t-test*.
