# Supplementary material for: Ectoderm to mesoderm transition by down-regulation of actomyosin contractility
Source: PLoS Biol. 2021 Jan 6;19(1):e3001060. doi: 10.1371/journal.pbio.3001060 (PMC7815211; doi:10.1371/journal.pbio.3001060)
Supplement: S4 Fig — (Related to Fig 4) (A–D) Rescue of Rnd1MO and ShiMO spreading and migration phenotypes. Four-cell stage embryos were injected in the dorsal side with COMO, RndMO, RndMO + YFP-Rnd1 mRNA (rescue), ShiMO, or ShiMO + YFP-Shirin mRNA (rescue). Dissociated mesoderm cells were plated on FN and time-lapse movies were recorded. The fourth condition represents RndMO or ShiMO cells treated with 50μM Y27632 Rock inhibitor (Y). Statistical comparions: 1-way ANOVA followed by Tukey HSD post hoc test. Red asterisks: Comparison to COMO. (E) Migration speed for different cell morphology categories. Analysis of data from Fig 4I. Red asterisks: comparison to COMO. One-way ANOVA followed by Tukey HSD post hoc test. Refer to S1 Data. ANOVA, analysis of variance; FN, fibronectin; HSD, honestly significant difference. (PDF) [file pbio.3001060.s006.pdf]

S4 Fig

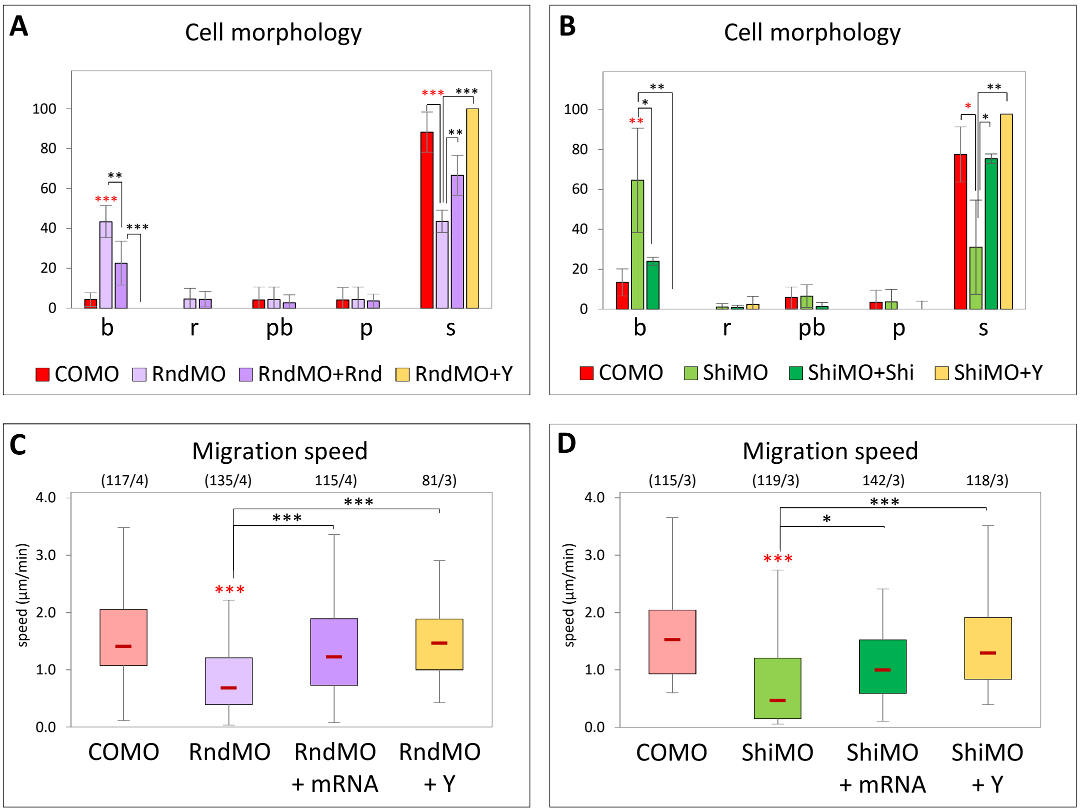

A-D) Rescue of Rnd1MO and ShiMO spreading and migration phenotypes (Related to Figure 4)**.** 4-cell stage embryos were injected in the dorsal side with COMO, RndMO, RndMO + YFP-Rnd1 mRNA (rescue), ShiMO, or ShiMO + YFP-Shirin mRNA (rescue). Dissociated mesoderm cells were plated on FN and time lapse movies were recorded. The fourth condition represents RndMO or ShiMO cells treated with 50µM Y27632 Rock inhibitor (Y). Statistical comparions: One-way ANOVA followed by Tukey's HSD post hoc test. Red asterisks: Comparison to COMO.

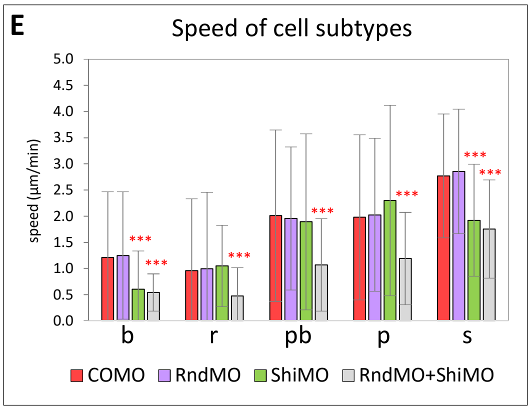

E) Migration speed for different cell morphology categories (Related to Figure 4). Analysis of data from figure 4I. Red asterisks: Comparison to COMO. one-way ANOVA followed by Tukey's HSD post hoc test.
