# Supplementary material for: Ectoderm to mesoderm transition by down-regulation of actomyosin contractility
Source: PLoS Biol. 2021 Jan 6;19(1):e3001060. doi: 10.1371/journal.pbio.3001060 (PMC7815211; doi:10.1371/journal.pbio.3001060)
Supplement: S5 Fig — (Related to Fig 5) (A) Morphometry of Rnd1 and Shirin induced spreading of ectoderm cells. The diagrams illustrate typical cell shapes. Corresponding images can be found in main Fig 5A–5E. These shapes were analyzed based on the following parameters: (A’) Area of the ventral contact surface (red in the schemes in A). (A”) Circularity of the ventral surface, which depends both on the roundness and regularity/convolution of the shape. (A”’) Ratio between the ventral area and the maximal cell area, calculated from maximal z projections. Refer to S1 Data. Blebs were excluded from measurements. Rnd1- and Shirin-expressing cells were here subdivided in 2 categorizes, low and high expression, based on the YFP fluorescence intensity. Note that these 2 categories overlap but are not equivalent Rnd1 expression levels had no significant impact on any parameter. Shirin expression had no effect on contact surface area, but high levels stimulated formation of convoluted protrusions (lower circularity) but decreased ventral/max area, reflecting the fact that many of them rounded up (fourth cell shape in panel A; see main Fig 5E). (B) Distribution of speed for ectoderm cells expressing Rnd1 or Shirin, compared to wild-type ectoderm and mesoderm. Brackets: range of high speed, comparable to mesoderm, achieved mainly by Rnd1-expressing cells. Refer to S1 Data. (PDF) [file pbio.3001060.s007.pdf]

S5 Fig

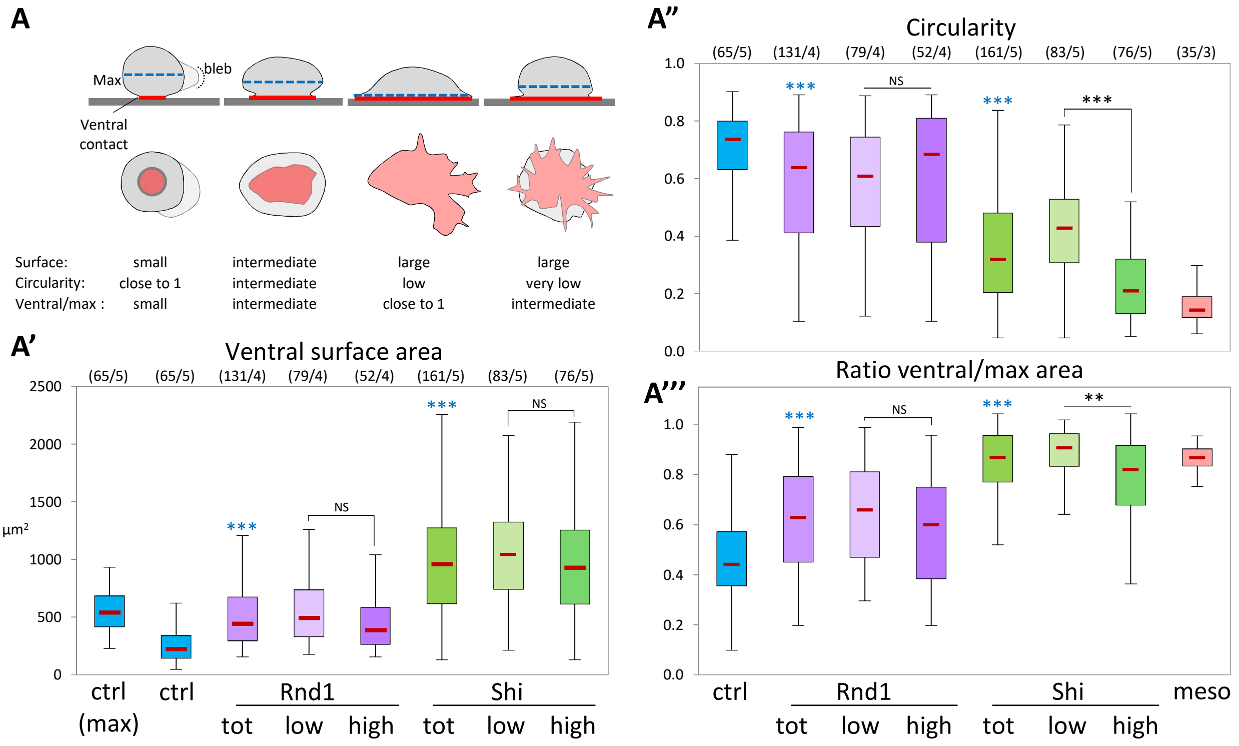

A) Morphometry of Rnd1 and Shirin induced spreading of ectoderm cells (Related to Figure 5).

The diagrams illustrate typical cell shapes. Corresponding images can be found in main Figure 5A-E. These shapes were analysed based on the following parameters: A') Area of the ventral contact surface (red in the schemes in A). A") Circularity of the ventral surface, which depends both on the roundness and regularity/convolution of the shape. A'") Ratio between the ventral area and the maximal cell area, calculated from maximal z projections. Blebs were excluded from measurements. Rnd1 and Shirin-expressing cells were here subdivided in two categorises, low and high-expression, based on the YFP fluorescence intensity. Note that these two categories overlap but are not equivalent Rnd1 expression levels had no significant impact on any parameter. Shirin expression had no effect on contact surface area, but high levels stimulated formation of convoluted protrusions (lower circularity) but decreased ventral/max area, reflecting the fact that many of them rounded up (4th cell shape in panel A, see main figure 5E).

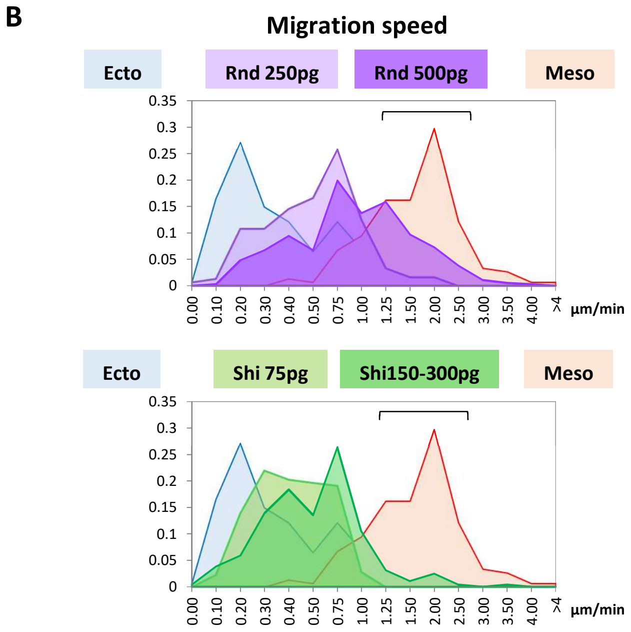

B) Distribution of speed for ectoderm cells expressing Rnd1 or Shirin, compared to wild type ectoderm and mesoderm (Related to Figure 5). Brackets: Range of high speed, comparable to mesoderm, achieved mainly by Rnd1-expressing cells.
