# Supplementary material for: Ectoderm to mesoderm transition by down-regulation of actomyosin contractility
Source: PLoS Biol. 2021 Jan 6;19(1):e3001060. doi: 10.1371/journal.pbio.3001060 (PMC7815211; doi:10.1371/journal.pbio.3001060)
Supplement: S6 Fig — (Related to Fig 6) Effect of Rnd1 and Shirin ectopic expression. (A) Loosening of ectoderm tissue upon expression of Shirin. Immunostained section of a YFP-Shirin expressing embryo showing a loosely organized ectoderm, characterized by the presence of large intercellular spaces (asterisks) and heterogenous β-catenin signal, weak signal along membranes except for strong local concentrations (arrowheads). Scale bar, 10 μm. (B–E) Cell orientation. (B–D) The main axis of deep ectoderm cells (double arrows) tend to orient roughly perpendicular to the inner surface of the tissue (dashed line). Rnd1-expressing cells show variable orientation. Shirin-expressing cells align parallel to the surface. Scale bars, 10 μm. (E) Quantification of the angle between the cell axis and the tissue interface. Numbers into brackets correspond to number of cells/embryos/experiments. Refer to S1 Data. (F–I) Analysis of β-catenin (green) and pMLC (red) in ectoderm explants. (F) Diagram, section of a control ectoderm explant (scale bar, 50 μm) and quantification. Statistical comparison using 1-way ANOVA followed by Tukey HSD post hoc test. (G–I) Examples of ectoderm explants. (G) β-catenin and pMLC signal along cell edges is highest in control (arrows). H,I) Explants expressing Rnd1 or Shirin. β-catenin tends to accumulate at cell vertices (concave arrowheads). pMLC levels are lower except for some cells (I, asterisks) that have rounded up and display high pMLC throughout the cell. Little to no β-catenin is seen between the round cells. Scale bars, 20 μm. (H) Effect of Rnd1 and Shirin expression on phosphorylation of MYPT. Dissected ectoderm tissues were analyzed by western blot. GAPDH was used as loading control, and the pMYPT signal was expressed as relative ratio, normalized to ectoderm control set to 1.0. Three independent experiments, statistical analysis using 1 sample, 2-sided t test. Refer to S1 Data. ANOVA, analysis of variance; HSD, honestly significant difference; MYPT, myosin light [file pbio.3001060.s008.pdf]

S6 Fig

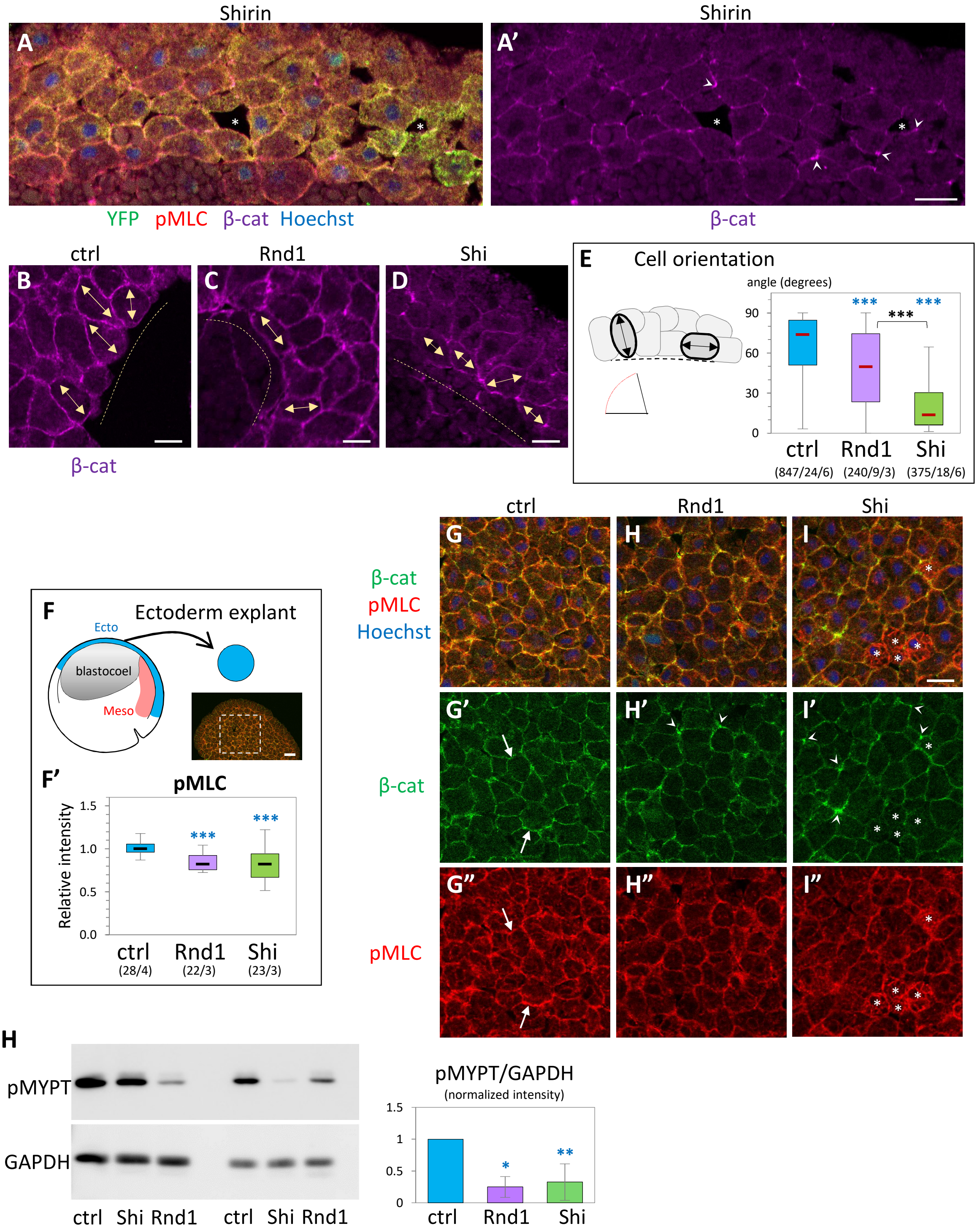

Effect of Rnd1 and Shirin ectopic expression (Related to Figure 6). **A) Loosening of ectoderm tissue upon expression of Shirin.** Immunostained section of a YFP-Shirin expressing embryo showing a loosely organized ectoderm, characterized by the presence of large intercellular spaces (asterisks) and heterogenous β-catenin signal, weak signal along membranes except for strong local concentrations (arrowheads). Scale bar, 10μm. **B-E) Cell orientation**. B-D) The main axis of deep ectoderm cells (double arrows) tend to orient roughly perpendicular to the inner surface of the tissue (dashed line). Rnd1-expressing cells show variable orientation. Shirin-expressing cells align parallel to the surface. Scale bars, 10μm. E) Quantification of the angle between the cell axis and the tissue interface. Numbers into brackets correspond to number of cells/embryos/experiments. Refer to S1 Data file.

F-I) Analysis of β-catenin (green) and pMLC (red) in ectoderm explants. F) Diagram, section of an control ectoderm explant (scale bar, 50μm) and quantification. Statistical comparison using one-way ANOVA followed by Tukey's HSD post hoc test. G-I) Examples of ectoderm explants. G) β-catenin and pMLC signal along cell edges is highest in control (arrows). H,I) Explants expressing Rnd1 or Shirin. β-catenin tends to accumulate at cell vertices (concave arrowheads). pMLC levels are lower except for some cells (I, asterisks) that have rounded up, and display high pMLC throughout the cell. Little to no β-catenin is seen between the round cells. Scale bars, 20μm. **H) Effect of Rnd1 and Shirin expression on phosphorylation of MYPT.** Dissected ectoderm tissues were analysed by Western Blot. GAPDH was used as loading control, and the pMYPT signal was expressed as relative ratio, normalized to ectoderm control set to 1.0. Three independent experiments, statistical analysis using one sample, two-sided *t-test*. Refer to S1 Data file.
