# Supplementary material for: Ectoderm to mesoderm transition by down-regulation of actomyosin contractility
Source: PLoS Biol. 2021 Jan 6;19(1):e3001060. doi: 10.1371/journal.pbio.3001060 (PMC7815211; doi:10.1371/journal.pbio.3001060)
Supplement: S7 Fig — (Related to Fig 9) (A) Example of ectoderm explant showing late partial spreading, which is only observed beyond the 120 minutes. (B) Examples of traces for single explants, illustrating the irregular expansion of mesoderm explants interrupted by retractions. In contrast, expansion of Y27632-treated mesoderm is smooth. (C) Quantification of average triangle size at the initiation of spreading (30 minutes) and the end of the time lapse (170 minutes). Refer to S1 Data. (PDF) [file pbio.3001060.s009.pdf]

S7 Fig

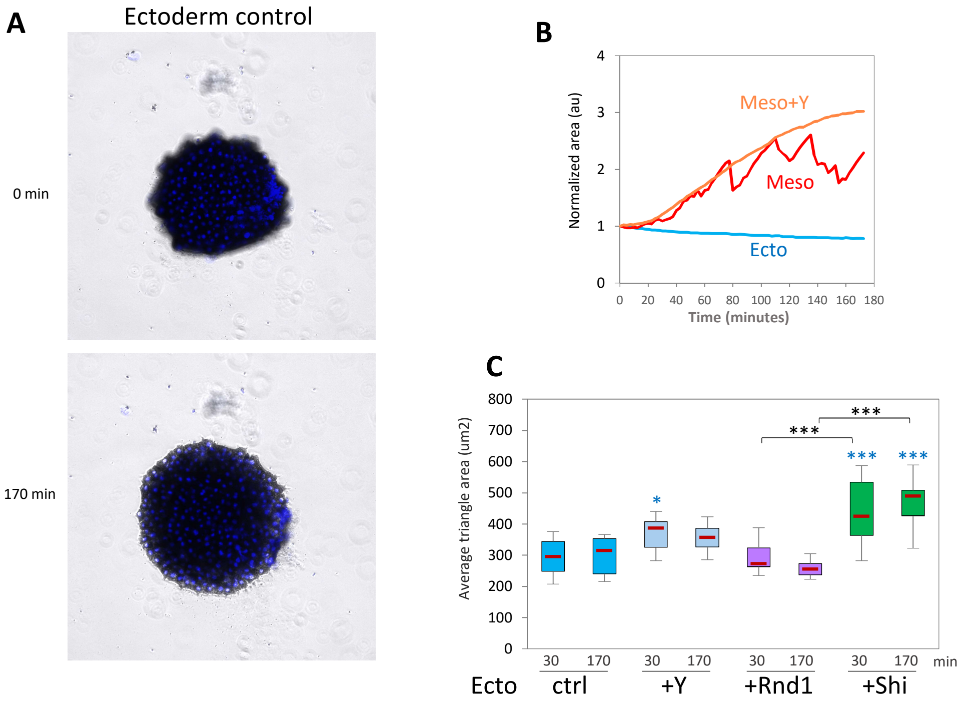

(Related to Figure 9): A) Example of ectoderm explant showing late partial spreading, which is only observed beyond the 120min. B) Examples of traces for single explants, illustrating the irregular expansion of mesoderm explants interrupted by retractions. In contrast, expansion of Y27632-treated mesoderm is smooth. C) Quantification of average triangle size at the initiation of spreading (30 min) and the end of the time lapse (170 min).
