# Supplementary material for: Ectoderm to mesoderm transition by down-regulation of actomyosin contractility
Source: PLoS Biol. 2021 Jan 6;19(1):e3001060. doi: 10.1371/journal.pbio.3001060 (PMC7815211; doi:10.1371/journal.pbio.3001060)
Supplement: S8 Fig — Analysis of spreading, dispersion, and intercalation of mesodermal explants under various conditions was performed as for experiments presented in Fig 9. (A–D) Control mesoderm, mesoderm treated with Y27632, and mesoderm from embryos injected with Rnd1 MO or Shirin MO. Red arrowheads in A and D indicate areas of large-scale retractions (compare 85 and 170 minutes). Scale bar: 100 μm. (E) Average time course curves with SD for the various experimental conditions. (E’) Corresponding relative spreading after 60 minutes and 170 minutes. (F–K) Delaunay triangulation of nuclei in order to measure cell dispersion. (F–I) Representative plots of triangulated nuclei after 170 minutes of imaging. X and Y labels mark the coordinates in μm, and the color-coded scale bar indicates the area of the triangles in μm2. (J) Quantification of average triangle size at the initiation of spreading (30 minutes) and the end of the time lapse. (K) Quantification of the relative change in triangle size over time calculated by dividing the average triangle area at 170 minutes by that at 30 minutes. (L) Quantification of intercalation calculated by dividing number of nuclei at the ventral surface at 170 minutes by the number at 30 minutes. Statistical comparisons: 1-way ANOVA followed by Tukey HSD post hoc test. Refer to S1 Data. ANOVA, analysis of variance; HSD, honestly significant difference; SD, standard deviation. (PDF) [file pbio.3001060.s010.pdf]

S8 Fig

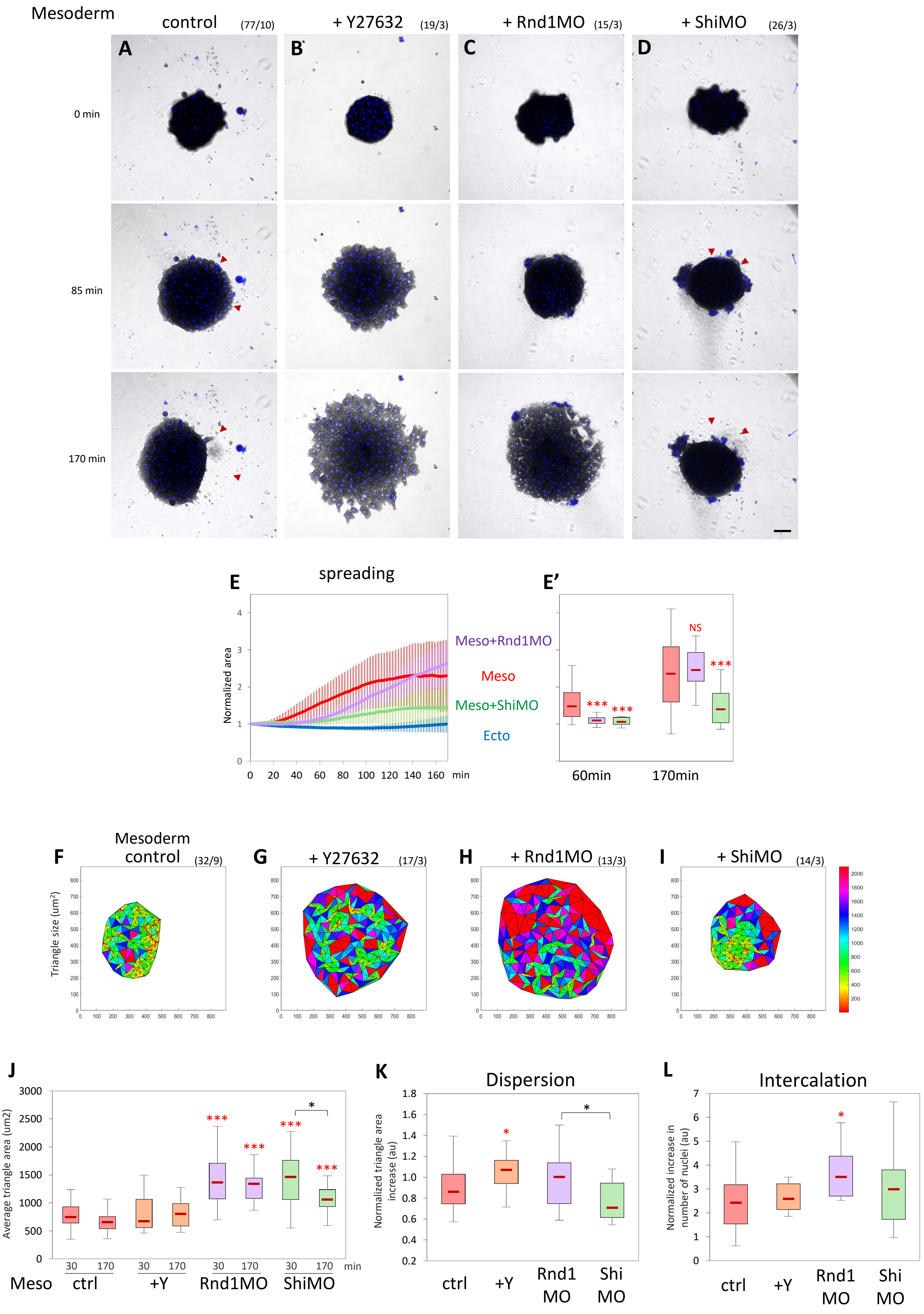
Mesoderm
control (77/10)
+ Y27632 (19/3)
+ Rnd1MO (15/3)
+ ShiMO (26/3)
A
B
C
D
0 min
85 min
170 min
E
spreading
Normalized area
Meso+Rnd1MO
Meso
Meso+ShiMO
Ecto
min
E'
NS
***
60min
170min
F
Mesoderm
control (32/9)
G
+ Y27632 (17/3)
H
+ Rnd1MO (13/3)
I
+ ShiMO (14/3)
Triangle size (um²)
J
Average triangle area (um2)
Meso
ctrl
+Y
Rnd1MO
ShiMO
min
K
Dispersion
Normalized triangle area increase (au)
ctrl
+Y
Rnd1 MO
Shi MO
L
Intercalation
Normalized increase in number of nuclei (au)
ctrl
+Y
Rnd1 MO
Shi MO

S8 Fig

Knockdown of Rnd1 or Shirin affect collective properties of mesoderm tissue explants (Related to Figure 9). Analysis of spreading, dispersion, and intercalation of mesodermal explants under various conditions was performed as for experiments presented in Figure 9. A-D) Control mesoderm, mesoderm treated with Y27632, and mesoderm from embryos injected with Rnd1 MO or Shirin MO. Red arrowheads in A and D indicate areas of large scale retractions (compare 85 and 170min). Scale bar: 100µm. E) Average time course curves with SD for the various experimental conditions. E') Corresponding relative spreading after 60min and 170min. F-K) Delaunay triangulation of nuclei in order to measure cell dispersion. F-I) Representative plots of triangulated nuclei after 170 minutes of imaging. X and Y labels mark the coordinates in µm, the colour coded scale bar indicates the area of the triangles in μm^2. J) Quantification of average triangle size at the initiation of spreading (30 min) and the end of the time lapse. K) Quantification of the relative change in triangle size over time calculated by dividing the average triangle area at 170 minutes by that at 30 minutes. L) Quantification of intercalation calculated by dividing number of nuclei at the ventral surface at 170 minutes by the number at 30 minutes. Statistical comparisons: One-way ANOVA followed by Tukey's HSD post hoc test.
