# Supplementary material for: Ectoderm to mesoderm transition by down-regulation of actomyosin contractility
Source: PLoS Biol. 2021 Jan 6;19(1):e3001060. doi: 10.1371/journal.pbio.3001060 (PMC7815211; doi:10.1371/journal.pbio.3001060)

S1 Appendix

Calculation of relative tensions and adhesiveness for cell doublets

Estimates of relative tensions were based on the principle that the geometry of the cell membranes at cell vertices reflects the equilibrium between the tensile forces exerted by the cell cortices [3, 16]. For a doublet formed of cell A and cell B, the equilibrium involved the cortical tensions at the two free cell surfaces (Ct_A and Ct_B) and the contact tension at cell-cell interface (T_{AB}). T_{AB} is defined as the sum of the cortical tensions of each cell at the contact (Ct_A' and Ct_B') and of the negative contribution due to cell-cell adhesion (-ω).

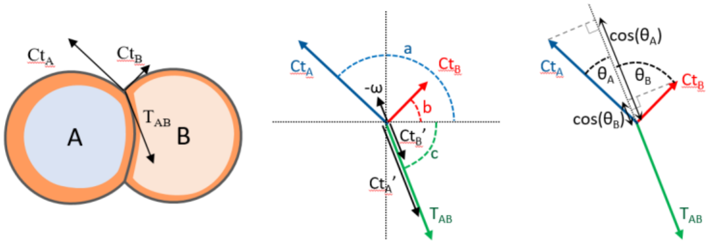

The force equilibrium was expressed by two equations:

(1) $$\sin(a) * Ct_A + \sin(b) * Ct_B + \sin(c) * T_{AB} = 0$$

(2) $$\cos(a) * Ct_A + \cos(b) * Ct_B + \cos(c) * T_{AB} = 0$$

Angles *a*, *b* and *c* corresponded to the orientation of each force vector.
Each of these angles was measured as the tangent to an arc fitted to three points of the cell membrane, including the cell vertex [3].

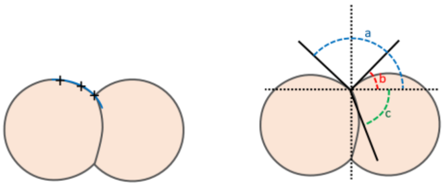

Based on equations (1) and (2), we could use the three angles to calculate the ratios between the two cortical tensions (Ct_A/Ct_B) and the ratio between contact tension and each of the cortical tensions [3] using the equation:

(3) $$T_{AB}/Ct_B = \left(\sin(b) - \frac{\sin(a) * \cos(b)}{\cos(a)}\right) / \left(\frac{\sin(a) * \cos(c)}{\cos(a)} - \sin(c)\right)$$

and the ratio Ct_A/Ct_B between the two cortical tensions:

(4) $$\frac{Ct_A}{Ct_B} = \left(\frac{T_{AB}}{Ct_B}\right) / \left(\frac{T_{AB}}{Ct_A}\right)$$

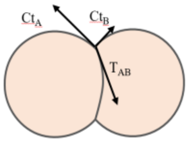

Ct_A/Ct_B is expected to be on average close to 1.0 for homotypic doublets. Ct_A/Ct_B of heterotypic provided a direct readout of the relative cortical tension of two cell types, here Rnd1 or Shirin-expressing cells compared to control ectoderm cells (Fig.8J, see below the complete results of all homotypic and heterotypic doublets). Note that Ct varies broadly even for cell of the same type/condition (e.g. homotypic control ectoderm, Fig.8J, see [3]).

To compare the tensions between different types of doublets, we calculated a relative contact tension *rel*T (T in the text and legends) expressed relative to the median value of control ectoderm contact tension set at 1. We also assumed that the average of the two cortical tensions $(Ct_A+Ct_B)/2$ was proportional to the median of the measured Cts. Thus, we defined a relative median cortical tension medCt for each condition, set to 1.0 for control ectoderm. *rel*T could then be calculated using the following equation:

(5) $$relT_{AB} = 2 * medT_A * \left(\frac{T_{AB}}{Ct_A}\right) / \left(1 + \frac{Ct_B}{Ct_A}\right) + 2 * medT_B * \left(\frac{T_{AB}}{Ct_B}\right) / \left(1 + \frac{Ct_A}{Ct_B}\right)$$

Adhesiveness α is an absolute number between 0 and 1, defined as the tension reduction at the contacts relative to free surface [19] and can be directly calculated via the contact angles [19]. We adapted this calculation to asymmetrical doublets, thus

(6) $$\alpha = 1 - \frac{T_{AB}}{Ct_A + Ct_B} = 1 - \left(\cos(\theta_A) + \cos(\theta_B)\right)/2$$

Note that adhesiveness does not depend on absolute Ct or T, but on the balance of both. Accordingly, as illustrated here below, two pairs of cells may have the same adhesiveness despite displaying very different cortical tensions.

Examples of configurations:

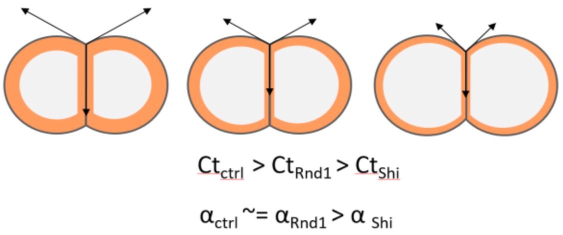

Complete results from doublet measurements:

Cortical tension, contact tension and adhesiveness of homotypic and heterotypic doublets of control ectoderm cells, and cells expressing Rnd1 or Shirin.

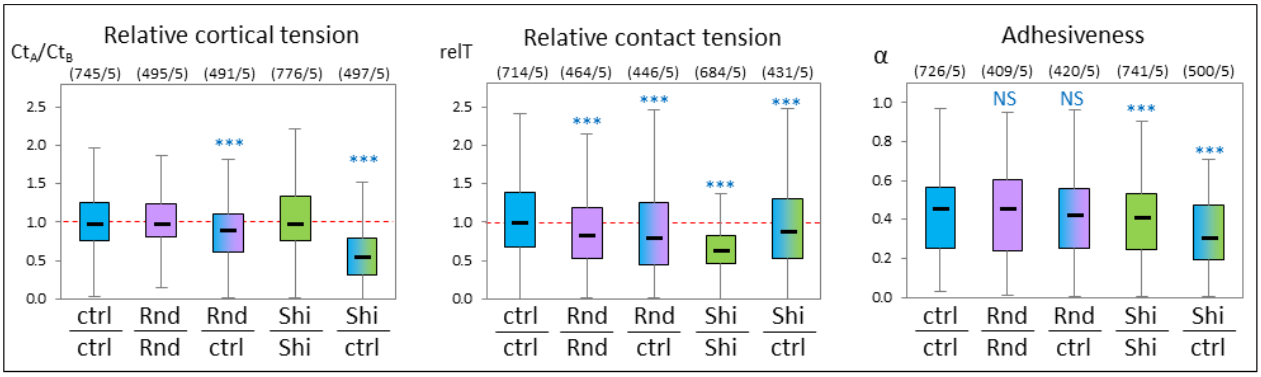

Supplement: S1 Appendix — (PDF) [file pbio.3001060.s011.pdf]
