# Supplementary material for: Ectoderm to mesoderm transition by down-regulation of actomyosin contractility
Source: PLoS Biol. 2021 Jan 6;19(1):e3001060. doi: 10.1371/journal.pbio.3001060 (PMC7815211; doi:10.1371/journal.pbio.3001060)
Supplement: S2 Raw Images — (PDF) [file pbio.3001060.s014.pdf]

S2 Raw Image

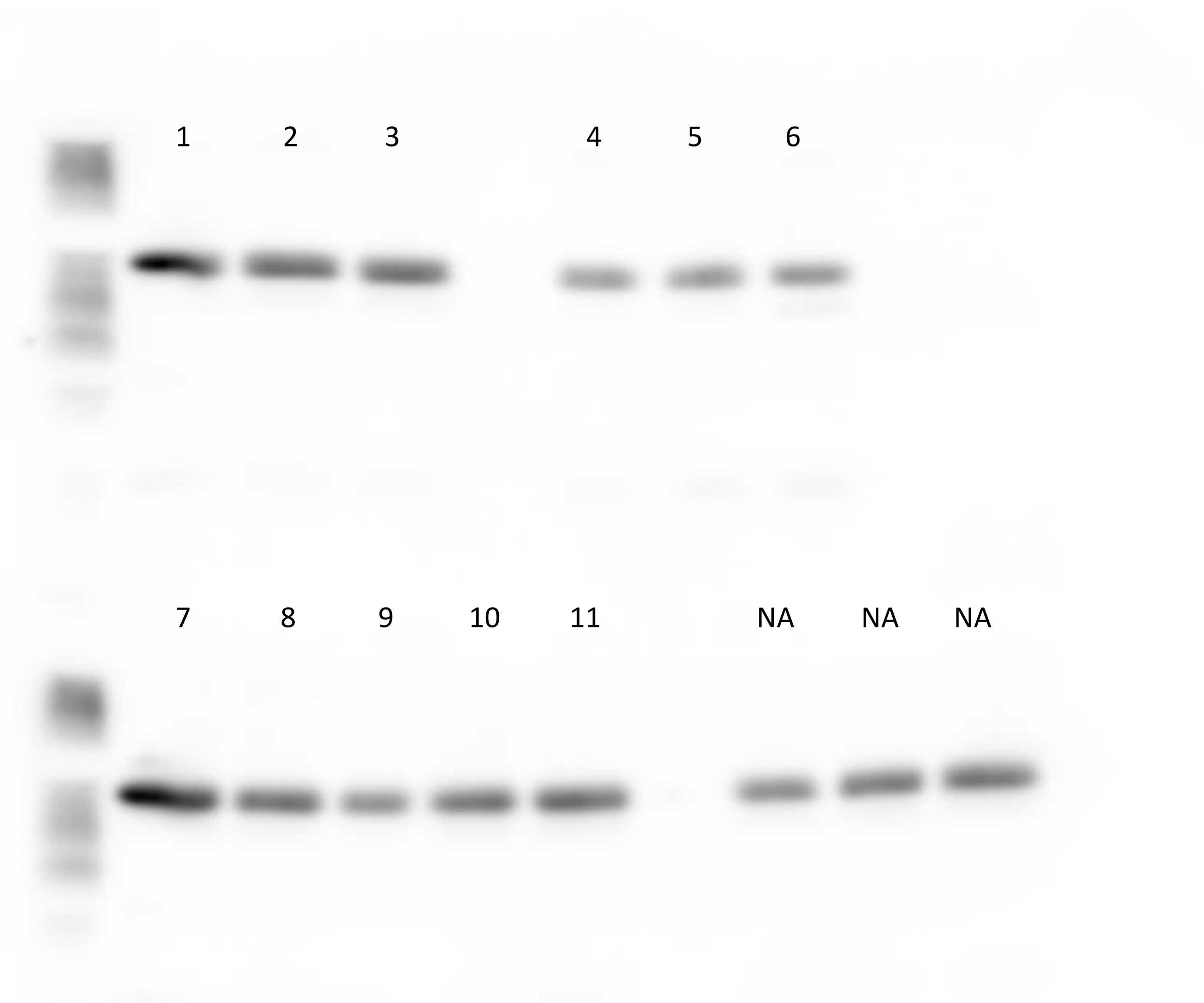

Western Blot for GAPDH (loading control) of ectoderm extracts (3 independent experiments). A dilution series of control of 3rd experiment (lanes 7-9) was used as standard for quantification. The numbered lanes correspond to the following conditions:

1) control; 2) + Shirin; 3) + Rnd1 (Experiment 1)

4) control; 5) + Shirin; 6) + Rnd1 (Experiment 2)

7) control (2x amount); 8) control (1x); 9) control (1/2 amount); 10) + Shirin; 11) + Rnd1 (Experiment 3)

NA: additional lanes not relevant for this study.
