# Supplementary material for: Ectoderm to mesoderm transition by down-regulation of actomyosin contractility
Source: PLoS Biol. 2021 Jan 6;19(1):e3001060. doi: 10.1371/journal.pbio.3001060 (PMC7815211; doi:10.1371/journal.pbio.3001060)
Supplement: Attachment — Submitted filename: Fagotto paper.docx.pdf [file pbio.3001060.s032.pdf]

The authors have addressed my comments satisfactorily and in my view the article is much improved by the additional experiments. Before publication, I have one remaining major issue that must be addressed. This is the relationship giving T_{AB}/C_{tB} (relationship (4)).

If I take the expressions (1) and (2) provided in the appendix and try to work out the relationships, I find something different from relationship (4).

Expression (2) can be rewritten to give C_{tA} as a function of T_{AB} and C_{tB}:

$$C_{tA} = -\frac{cosb}{cosa}C_{tB} - \frac{cosc}{cosa}T_{AB}$$

If we plug this expression into (1), we get:

$$-sina\frac{cosb}{cosa}C_{tB} - sina\frac{cosc}{cosa}T_{AB} + sinb.C_{tB} + sinc.T_{AB} = 0$$

Which can be rearranged as:

$$\left(sinb - sina\frac{cosb}{cosa}\right)C_{tB} + \left(sinc - sina\frac{cosc}{cosa}\right)T_{AB} = 0$$

And finally:

$$\frac{T_{AB}}{C_{tB}} = \frac{sinb - sina\frac{cosb}{cosa}}{sina\frac{cosc}{cosa} - sinc}$$

Which is different from (4) in the appendix. My guess is that (4) is in fact T_{AB}/C_{tA}. There is also an alpha that appears in their relationship, which I guess is a typo.

The authors should recheck their appendix and reevaluate everything that derives from (4). It would also be much clearer if they used some equation editing software – like Latex or the word equation editor. Relationship (6) is very difficult to read and could be simplified by noting that $(T_{AB}/C_{tA})/(T_{AB}/C_{tB})$ is in fact C_{tB}/C_{tA}.
