# Supplementary material for: Ectoderm to mesoderm transition by down-regulation of actomyosin contractility
Source: PLoS Biol. 2021 Jan 6;19(1):e3001060. doi: 10.1371/journal.pbio.3001060 (PMC7815211; doi:10.1371/journal.pbio.3001060)
Supplement: Attachment — Submitted filename: reviewer response-20200918 final.docx [file pbio.3001060.s031.docx]

We would like to thank the reviewers for their constructive criticisms and for the suggested improvements, which we believe have further strengthened the coherence of this study. Please find here below the point by point detailed answers to the comments.

Rev. 1:

In this paper, Kashkooli et. al. provide compelling evidence that the ectoderm to mesoderm transition in gastrulating Xenopus embryos involves downregulation of actomyosin contractility. Furthermore, they show this reduction in contractility is driven by two negative regulators of RhoA, namely Rnd1 and Shirin. Some experiments were performed in intact embryos, but most of the experiments assayed the morphology and migratory behavior of explants or isolated cells when plated on sparse FN in vitro. Mesoderm and ectoderm show distinct morphologies: ectoderm are round and compact with a stable ring of adhesions and blebs, while mesoderm are spread, protrusive, with dynamic adhesions and are more migratory. Acute treatment with Rock inhibitors causes the ectoderm to behave more similar to mesoderm, except that it migrates slower. Rnd1 and Shirin are more highly expressed in the mesoderm. Morpholino knockdown of either resulted in gastrulation defects, with a block in mesoderm involution. In vitro, mesoderm KD for Rnd1 or Shirin look like ectoderm, and the phenotype can be rescued by Rock inhibition. However, differences in cell-matrix adhesion patterns and migration rates were observed between Rnd1 and Shirin MO. Overexpression of Rnd1 or Shirin in ectoderm cells rendered them mesoderm-like. Rnd1 localizes to the cortex and Shirin to cell-matrix adhesion and protrusions. Micropipette aspiration assays documented the changes to the physical properties of the mesoderm and ectoderm tissues after contractility inhibition by a drug or Rnd1/Shirin expression in ectoderm.

Overall, I found the paper to be clear, well executed and controlled, and intriguing. I did not find any major problem that should preclude it from being accepted for publication. Below is a list of minor issues I recommend addressing before the actual publication:

1. Stars of statistical significance need to be on a comparison, not on a single column.

We now reverted to the standard convention of statistical comparisons with black stars positioned on top of brackets works for the simplest graphs (e.g. Fig.1). However, for more complex graphs, this representation is heavy and difficult to read, especially when multiple conditions must all be compared to control ectoderm or control mesoderm. This would require to superimpose multiple lines/brackets. We find that the possibility to use colours is an opportunity to clarify the presentation of these data. We thus use a consistent colour code for the columns/boxes and for the corresponding statistical comparisons (blue star for comparison with the blue column of control ectoderm, red star for comparison with the red column of control mesoderm). We do use black asterisks and brackets for additional comparisons between specific conditions. We have made sure to clearly explain the colour code in the legend of figure 1, and then use it consistently for all figures.

2. Figure 1, why is D' not the same cells as D?

D’ was an enlargement of top part of D, we have now added the outline of the region in D.

3. When describing the results of the adhesion assay by rotation the authors write about ectoderm binding to FN that they "adhered almost as efficiently as mesoderm cells" and when describing their adhesion to cadherin they write "Ectoderm cells showed significantly higher cadherin adhesion than mesoderm cells". However, in both cases the difference is modest and yet statistically significant and so should be described the same way.

We have modified the description of the result of adhesion on FN.

Note, however, that the difference of adhesion on cadherin between ectoderm and mesoderm is of about 30%, which cannot be viewed as modest.

We would like to explain here why we presented in a different perspective adhesion on cadherin and on FN:

Cadherin adhesion had been already investigated, and the results of our assay are consistent with other previous data.

The situation was different for FN: Adhesion on FN had not yet been investigated, but the well-known fact that ectoderm cells “failed” to spread on FN had led to the general assumption that adhesion to this matrix was very poor. Our results do show that mesoderm adheres better than ectoderm, and indeed the amplitude of the difference (~25%) is as significant as the opposite difference on cadherins. Nevertheless, considering the previous assumption, ectoderm adhesion is surprisingly robust. We thus would like to convey the message that this adhesion (and the corresponding adhesive ring structure) is an unexpected discovery.

4. Fig.1C', yellow concave arrows - there are no yellow concave arrows in 1C'. Maybe meant yellow arrows in 1C?

Yes, indeed. Now corrected.

5. Fig.1D', white and yellow concave arrows - should be fig1D?

In both, indeed, now corrected

6. The authors write: "In all these aspects, Rock inhibition appeared sufficient to induce a dramatic transformation of ectoderm cells into mesoderm- like cells." - It should be noted, as seen in Fig2G, that the migration speed is still far from mesoderm.

Yes, this important note has been added.

7. Regarding the experiments with whole embryos the authors write: "While there were differences between the detailed phenotypes of the two knockdowns, their analysis was of little informative value toward understanding the underlying mechanisms." - why? What about the double KD?

Yes, the sentence was rather unclear. We actually meant that a phenotype as dramatic as the one we observed is difficult to interpret mechanistically in the context of the whole embryo. The possibility to isolate tissues and cells and use a panel of assays is thus particularly powerful to learn more about this process. But this note was actually unnecessary, and we decided to remove from the revised manuscript.

About the double KD, there is not much more to be learnt from it, since gastrulation is already blocked with a single KD. The double KD is only mentioned for migration assays, because in single KDs some migration was still observed. Yet, apparently this residual migration is not sufficient to drive involution in the embryo. We did not further explore double KDs for two reasons: Firstly, the severity of the single KDs made double KD unnecessary. Secondly, double-depleted tissues were too strongly affected to permit proper explanation and analysis (difficulty to dissect, very poor explant “healing”).

8. In figure 3 in the graphs F, G there is a condition of Rnd1MO + ShiMO - this condition is not mentioned in the results text.

Now included (note that Fig3 is now figure 4).

9. Shirin has strong effect on cortical tension and cell spreading while rnd1 has mild effect. However, Rnd1 expression in the mesoderm is much stronger relative to ectoderm compared to shirin. The authors could discuss why this might be the case.

We briefly discuss this stronger activity of Shirin (at least for some of the cell and tissue properties), which may be due to its direct catalytic GAP activity, while Rnd1 acts indirectly (via p190GAP). (Discussion, last paragraph of page 12)

10. "From the comparison of the effects of Rnd1 and Shirin on tension (Fig.5J) and on cell spreading (Fig.4G), we can extrapolate that a ~20% reduction in cortical tension may be sufficient to allow ectoderm cells to start to elongate and spread." - Not clear how this extrapolation was done.

We have considered explaining the reasoning for this estimate, but eventually we came to the conclusion that it was too hypothetical and removed it from the text.

11. "For this purpose, we dissected tissue explants, let them heal," - what does that mean? How long does the healing process take?

We now explain if briefly in the text as well as in the materials and methods: Explants spontaneously round up to form a sphere. This takes about 30min, we leave them 45 min to insure complete “healing”.

Rev. 2:

This manuscript investigates how the differences in morphology and motility between mesoderm and ectoderm are controlled in Xenopus. The central hypothesis of this paper is that these differences arises from differences in the expression of regulators of Rho-kinase. This is an important topic as the control of mechanics in the different germ layers at the cellular level will play a key role in tissue morphogenesis. It is also interesting that mechanical differences are controlled by only few proteins. The experiments are interesting and generally well quantified using a muli-disciplinary approach. Overall, I have only few issues with the data and most can be resolved with image analysis. Following revisions, I recommend publication.

Major issues:

-page 9, paragraph 1: the authors state that shirin expressing explants become looser with many cells migrating away whereas rnd1 explants spread while remaining compact. This is an important result and it would be good to quantify this more carefully. Maybe by looking at a triangulation of the cell centroids and the average area of the triangles over time as others have done for neural crest? Or by quantifying the distribution of numbers of neighbours over time?

This excellent suggestion brought us tremendously useful quantitative information about the properties of the tissue and the effects of the regulators. We have now included a complete analysis by Delaunay triangulation for all the conditions. The figure has become quite large and has been split into a main Figure 9 and supplementary Figures S7 and S8.

As presented in the corresponding result section, triangulation data gave us three types of information:

- Firstly, we could differentiate between “cohesive” and “dispersive” migration, simply by monitoring the change in average triangle areas (thus reflecting the distance between nuclei). As explained in the result, a cohesive migration is expected to maintain constant areas, while dispersive migration results in increasing areas. Consistently, the ratio between a late and an early time point (170min and 30min, see figure legends for rationale of choosing these points) remains close to 1 for control ectoderm, but is significantly increased by Shirin expression, unambiguously confirming that the cells indeed dispersed. Furthermore, we made the striking observation that Rnd1 decreased the area (<1), indicative of “compaction” of the explant. Consistently, Shirin depletion in the mesoderm also led to a ratio <1, supporting our interpretation that depleting Shirin tilted the balance toward the action of Rnd1, thus toward a more cohesive mode of migration.

- A second information came from the analysis of the first time points, which provided indications about the initial compaction of the explants, which were completely consistent with what we observed while dissecting and manipulating the tissues, and in agreement with all our other data: Shirin explants were indeed looser from the very beginning, while Rnd1 explants were similar to control ectoderm.

- Segmentation of all nuclei at the ventral surface of the explant, which was necessary for the triangulation analysis, also provided the opportunity to calculate the number of nuclei being added over time to this basal layer, thus providing a quantitative measure of intercalation, another key parameter of mesoderm morphogenesis. We thus extracted another very interesting set of data, which highlighted in particular the remarkable capacity of Rnd1 to stimulate massive intercalation.

Note that the interpretation of data on MO conditions is generally less straightforward than that of the ectopic expression experiments. Depletion of either Rnd1 or Shirin must lead to an imbalance between these two activities, which in some aspects appear to be additive, in others complementary, and potentially even antagonistic.

-Do the authors have western blots showing that in Rnd1 or Shirin expression in ectoderm explants decreases the level of RhoGTP or pROCK compared to control explants? Given the current circumstances, if the authors have not acquired this data, it is ok to ignore this comment. But if they do have it, it would be nice to add it because it will make the role of Rnd1 and Shirin as regulators of Rho-ROCK signalling during morphogenesis more convincing.

We present in suppl. Fig.S6H a Western blot showing that expression of Shirin or Rnd1 decrease phosphorylation of myosin light chain phosphatase, an established direct target of Rock, in ectoderm explants.

-page 6, last paragraph: the authors state that shirin expression caused cells to overspread. The authors should quantify cell spreading. This would be a good complement to the morphological classification in panel G.

This suggestion was very useful. We went back to our images and performed a careful morphometric analysis, now included in supplementary Fig.5. We measured the total ventral (contact) surface area, the circularity (which reflects both elongation and convolution of this surface, thus also formation of complex protrusions). We also measured the ratio between the ventral area and the maximal area projection, which gives a quantitative read-out for cell spreading. This analysis has refined the differences between the Rnd1 and Shirin phenotypes. It has also allowed us to highlight the fact that strong overexpression tended to make the cell round up. Thus, those cells that we had described as “overspread” are in fact rounder than other Shirin-expressing cells, even though they have more expanded and complex protrusions. Note that high Rnd1 also tends to make cells round up and dissociate. This distinction between low and high-expression phenotypes is also useful to explain why we find that these regulators have a promigratory role during gastrulation, while in other studies, these molecules were presented as antiadhesive and antimigratory.

-Could the authors give more details about the calculation of the various mechanical parameters they extract from the doublet experiments? An appendix would be helpful for the readers and make the paper self contained. It would also allow me to understand exactly what the authors have done, which I find a bit difficult to judge currently.

The charts in the main figure, which is now figure 8, have been simplified, which should make the key results easier to grasp. A more detailed explanation of the calculations has been included as an appendix along with the non-abridged charts.

Minor issues:

-In some of the figures, the control is the mesoderm (e.g. Fig 3), in others it is the ectoderm (e.g. Fig 4). The authors have tried to colour code this but there are so many colours involved in the graphs that it is difficult to remember what colour represents what. On the legend, they just write COMO or ctrl. Could the authors also add the name of the germ layer (Como meso or ctrl ecto)?

We have followed the reviewer’s suggestions and specified for each experiment whether the control is ectoderm or mesoderm.

-In the discussion, paragraph 3, the solid to liquid transition for biological materials is not an all or nothing transition. There is a whole spectrum of properties that are more solid-like or more liquid-like.

We agree with the reviewer, and we have clarified this part of the discussion. We think that the ectoderm and mesoderm exist very close to each other on the continuum of viscoelastic properties.

Rev. 3:

Ectoderm to mesoderm transition is a fundamental process in embryogenesis, leading to the transition of resting epithelium to moving mesodermal cells. Kashkooli and co-workers here provide compelling evidence that the inhibition of the Rho/ROCK axis is sufficient to induce a mesenchymal-like motile behavior in isolated and grouped epithelial cells. Using expression analysis and morpholino-based interference, the authors show that the Rho inhibitors Rnd1 and Shirin are both upregulated in mesodermal cells and that downregulation of both reverts the phenotypic switch and adhesion to fibronectin and cadherin ligand in vitro, and perturbs gastrulation in vivo. Rnd1, but not Shirin, was required for focal adhesion formation, whereas Shirin preferentially supported speed gain. Thus, Rnd1 and Shirin, by regulating Rho, critically contribute to ectoderm-to-mesoderm transition.

The concept that lowering Rho activity induces epithelial cell kinetics as the onset for mesoderm migration is compelling, and that Rnd1 and Shirin control Rho in this process is functionally worked out well. The multicellular explant culture delivers a reliable model for quantifying cell kinetics and dispersion. However, by which mechanism Rho regulation initiates this transition, directly vial actomyosin regulation or via affecting cell-cell junctions, and how Rnd1/Shirin differentially regulate this complex process, independently or in series, remain unclear.

In addition, mechanistic key claims are based on in vitro findings, using isolated cells monitored in 2D culture systems in short-term culture (few hours), whereas in vivo validation was only performed for global endpoint analysis. It remains unclear how the identified phenotypes and derived conclusions hold up when more thoroughly compared to in vivo analyses. Lastly, while the data show that Rnd1/Shirin are required to initiate gastrulation, it is not clear whether they are sufficient to trigger gastrulation in vivo.

Before responding to the specific comments of reviewer 3, we would like to clarify a few points:

1. Mesoderm internalization during gastrulation is a complex process, which we do not aim to solve in a single paper. What we address here is the fundamental difference between motility STATES between ectoderm and mesoderm. Motility is the very basic requirement for mesoderm movement. It does not define a particular stage of gastrulation, but is a property maintained throughout gastrulation, as the mesoderm progressively migrates along the blastocoel roof (the inner ectoderm surface), while it also thins through the process of radial intercalation.

2. Along the same lines, this study does not address the “initiation” of gastrulation, and not even initiation of mesoderm internalization: Mesoderm motility is required, but it clearly cannot alone “create” gastrulation. Without going into the details of whole process, one may mention that endoderm vegetal rotation and invagination of endoderm bottle cells are essential to direct the inwards movement of the mesoderm and bring it into contact with the blastocoel floor. Mesoderm motility should be viewed as the MOTOR of its internalization movement, but other parts are need, (steering wheel, gears,…)

3. Mesoderm involution involves both migration along the ectoderm roof and intercalation. The ectoderm roof acts as substrate BOTH through secreted fibronectin and through cadherin adhesions. Intercalation requires cell moving relative to each other, which is mostly using cadherin-based adhesion as support.

Thus, one major point of our study is the discovery of the principle that confers mesoderm with cell motility through decrease in cortical contractility. Motility, in turn, is necessary for migration both on matrix and on other cells.

The second discovery is the identification of two regulators which share the capacity to activate this motility, but also display very interesting complementary properties.

1. In vivo, both ectodermal and mesenchymal states retain cell-cell junctions, enabling either cohesive epithelial barrier formation or collective invagination and mesoderm invasion. However, most in vitro results were derived using fully isolated cells, which disregards that cell-cell junction regulation is involved in gastrulation. How is the initiation of gastrulation represented by the cell-substrate (FN) interaction of isolated cells? Is indeed a focal adhesion formation required for initiating or maintaining gastrulation? Can the morphological and adhesion features, and the speed data more precisely be linked to the in vivo phenotype? The significance of the mechanistic conclusions could be strengthened by performing in vitro and time-lapse in vivo analyses side-by-side to validate at least key points on cell function for the multicellular process in vivo.

Regarding FN adhesion, we have cited additional work that shows that FN based adhesions are important for migration of the mesoderm along the blastocoel roof. We have also emphasized that the most fundamental aspect of gastrulation is cell motility, and that, while recognizing their limitations, the single cell experiments were critical first steps in establishing that we were able to induce this basic trait in the normally non-motile ectoderm. These experiments set the stage for the explant experiments, where we first showed that we could reproduce two further key traits of gastrulation, namely collective cell migration (spreading and migrating of the explant), and intercellular migration (intercalation). We also include a discussion of the limitations of single cells and how their properties relate to higher levels of organization (i.e. tissues and whole embryo) on end of page 13 and page 14.

About the analysis of cell-substrate versus cell-cell interactions:

a) At the single cell level, we have:

- tested adhesion systematically in both contexts, using FN and recombinant cadherin as substrates.

- systematically studied subcellular localization of myosin, Rock, Rnd1 and Shirin at both matrix and cell-cell contacts

- vinculin is used as a readout of stress, as it is a well-established mechanosensory protein

- vinculin does not accumulate at cell-cell contacts, probably because forces are below the threshold required for its recruitment. Yet, we had a better assay to study cell-cell adhesion, i.e. the measurement of cell doublets, which provides precise and unambiguous quantitative relative data about the tensions exerted by these cell contacts and the resulting adhesiveness

-note that in terms of single cell migration, FN is used as standard substrate in the field. We have not been able to make cells migrate on immobilized recombinant cadherin, although we are currently working intensively to achieve this goal.

b) At the tissue level (explants):

- Our tissue spreading assay provides a powerful readout for collective migration. Of course, this migration results from the complex balance of cell motility, adhesion to matrix, and adhesion to other cells. These are actually the very same parameters at work in the in vivo situation, although obviously, the balance is different.

- Note that our new detailed analysis of collective migration, as suggested by reviewer 2, has also allowed us to quantify radial intercalation, thus adding an important aspect of mesoderm migration to our analysis.

- The pipette aspiration assay provides precise information about tissue properties in the absence of FN substrates. Here, tissue properties are exclusively dependent on internal cell properties (cytoskeleton) and cell-cell contacts.

c) Embryo level: We now provide a complete quantitative immunofluorescence analysis of cell-cell contacts (β-catenin) and myosin activity (pMLC) at the cell cortex in whole embryos, comparing wild type ectoderm and mesoderm tissues with ectoderm ectopically expressing Rnd1 or Shirin. These new data confirm the results obtained with the multiple other assays performed at the single cell and tissue explant level, further strengthening the relevance of all our assays and the coherence of our results.

About migration kinetics:

The reviewer may be aware that live microscopy imaging of the mesoderm within the whole Xenopus embryo is not possible. Nevertheless, speed of migration of the endogenous mesoderm can be estimated from fixed samples of consecutive stages, and it advances at a speed of about 2μm/min (we now cite R. Winklbauer, personal communication, but a rough estimate can be done by anybody on published images of whole embryo sections, comparing advance of mesoderm relative to the blastopore lip at different developmental stages, and this is indeed the speed that can be obtained).

In our migration assay, the speed of single mesoderm cells is ~ 1.5-2.5μm/min.

In our explant spreading assay, expansion of mesoderm explants occurs at about 1μm/min.

These values are strikingly close, further validating the physiological relevance of our in vitro assays.

We now mention these estimates in the discussion.

2. The roles of Rnd1 and Shirin were tested using morpholino-based downregulation, and this resulted in defective cell-substrate interaction and migration in vitro. However, it is not clear whether both regulators mediate gastrulation, and whether their induction suffices to induce mesoderm migration. Is ectopic expression of YFP-Rnd1 or YFP-Shirin sufficient to induce gastrulation in multicellular ectodermal explants and in vivo?

We believe that the whole embryo phenotypes following morpholino knockdown of either Shirin or Rnd1 demonstrate clearly that removal of either is enough to completely block gastrulation. We have now moved this data to Figure 3 in the main text.

We also think that it is clear from both figures 4 and 9 that the ectopic expression either shirin or Rnd1 is sufficient to induce spreading and migration in both single cells and tissue explants, though to a lesser degree than control mesoderm.

In terms of inducing gastrulation, in vivo induction of gastrulation in the ectoderm would not be possible, as gastrulation movements are extremely dependent on specific tissue/mechanical contexts. For instance, the vegetal rotation of the endoderm and the initial invagination caused by the formation of the bottle cells, which initially bring the mesendoderm into contact with the blastocoel roof. However, we have now included immunofluorescence data on whole embryos injected with Rnd1 or Shirin that show both regulators do impact the morphology of the animal cap. These data are consistent with the proposed function of these regulators (Fig.6 and S6).

Though some morphogenetic processes during gastrulation are tissue autonomous, it is hard to constitute what qualifies as gastrulation in different explant systems. The explant system we used in this paper do not gastrulate, however, the isolated prechordal mesoderm used in the mesoderm experiments is still capable of radial intercalation, a key process in gastrulation. We have now quantified intercalation in Fig.9N and S8L, and we show that both Shirin and Rnd1 are capable of inducing intercalation, though Rnd1 does this to a much greater degree, consistent with other data suggesting it is pro-adhesive.

3. How much Rho silencing is required to induce epithelial dynamics, and how much Rho activity is required to maintain mesenchymal movement? There is a potential fine line between both requirements. How effective was the Rho/ROCK inhibition in either setting, including knockdown experiments? Biochemical (if feasible) or in situ analysis of the ROCK inhibitor effects should be provided, such as pMLC/MLC stainings after molecular intervention in vitro and in vivo. Which level of Rho/ROCK activity is required to maintain mesoderm migration?

Indeed, the question of the balance of positive and negative functions of Rho in adhesion and motility is a very interesting question. We have now included immunofluorescence experiments that quantify the levels of pMLC both in the whole embryo (Fig.6) and in ectoderm explants (Fig.S6F) after ectopic expression of Rnd1 or Shirin. We have also included biochemical analysis via western blot of phosphorylation of the regulatory subunit of myosin light chain phosphatase (MYPT) which is a direct target of ROCK1/2. Following ectopic expression of Rnd1 and Shirin both pMLC and mMYPT levels were decreased.

We also simplified the doublet analysis in figure 8 to make the main conclusions clearer. Relevant for this point, the fact that both Rnd1 and Shirin decrease cortical tension of ectoderm cells is reflected in the TST values measured for explants in figure 10. We recognize that these experiments focused on ectoderm ectopic expression, but this was due to the much clearer and easier to interpret nature of these gain of function experiments compared to the more complicated phenotypes of the knockdowns in mesoderm. Not to mention the difficulty working with the morphant mesoderm tissues as they were so powerfully impacted by the knockdown.

We did attempt to estimate the decrease in cortical tension that would be required to stimulate spreading and migration of the ectoderm. Based on Fig.5G, >95% of wild type ectoderm cells adopt a round blebbing morphology when plated on FN, and <1% of them show signs of spreading (p and b categories). The distribution of normalized Ct for control ectoderm homotypic doublets indicates a lower quartile at 0.8. We can thus conservatively conclude that Ct of ectoderm cells can fluctuate of at least +/- 20% without causing their spreading. On the other hand, about 50% of Shirin-expressing ectoderm cells spread (60% including partial spreading of the p category). Heterotypic doublets indicate that the median relative Ct of these cells is 0.55, and 60% of them have a relative Ct lower than 0.65. As for Rnd1-expressing cells, 35-40% spread (Fig.5G), their median Ct is about 10% lower than controls, and 40% of them have a Ct lower than 0.8. Taken together, these data indicate that the threshold for cell spreading can be set between 20-40% reduction in Ct. However, we felt that this was too speculative to include in the manuscript, and that further experimental validation was beyond the scope of this study.

4. Descriptors/measures of ectodermal-to-mesoderm transition are not well described and consistently used throughout the manuscript. While gain of invasiveness (migration speed, cell area) and single cell dispersion (cohesion) are features of the mesodermal state, these parameters are not consistently quantified after molecular interference. As a consequence, we are left uncertain which overlapping and distinct roles of Rnd1 and Shirin on cell-cell cohesiveness and substrate adhesion underlying the transition in individualized cells, multicellular explants and in vivo are.

We have added additional quantifications of cell area/spreading (figure S5) and dispersion (Figure 9 and S6) to the manuscript. With respect to migration speed, single cell migration speed is consistently quantified. For the analysis of explant spreading, we found that measuring explant area over time was more informative than measuring cell migration of the cells within the explants as spreading gives an indirect measure of cell migration as well as revealing other interesting characteristics (e.g. phases of spreading and contracting). Initial measurements of cell migration speed in the explants yielded the exact same trends as the spreading analysis so we decided not to continue measuring it. We emphasize how similar the rates of migration are across all assays, also relative to migration in the embryo in the discussion (end of paragraph 1, page 14).

The immunostaining experiments on whole embryos, with quantifications of β-catenin as a marker for cell adhesions (Fig.6, Fig.S6) were consistent with other data on cell-cell adhesion found in Fig.4H, Fig.5K, Fig.8L, Fig. 9M (new), Fig.S8K (new), and Fig.10EH. Though these experiments all use different approaches, they all provide information on cell-cell adhesion - some more direct than others – that is consistent across the different experiments. We believe that this diversity in approach is in fact a strength of the study.

We also emphasize the consistency of our results in several locations of the manuscript (e.g. last paragraph page 8, last line paragraph 1 page 9). In addition to being clearer about the diverging activities of Rnd1 and Shirin throughout the results (e.g. paragraph 2 and 3 page 6, paragraph 2 page 11) we include a more detailed discussion of their differences on page 12 and 13.

5. Rho silencing along cell-cell junctions is an important mechanism to support cell-cell cohesion, so their interventions are expected to affect both cell-substrate and cell-cell adhesions. The authors focus on actomyosin contractility and substrate adhesion, but neglect the role of Rho on cell-cell junction regulation. How is the viscosity regulation shown in Fig. 8 linked to adherens junction stability and cortical actin organization along adherens junctions? How are cell-cell interactions perturbed by Rnd1 and Shirin regulation and how critical are they in permitting ectoderm-to-mesoderm transition?

We discuss this point in the response to point 4, but to repeat: we included in vivo immunostaining experiments with quantifications of β-catenin as a marker for cell adhesions (Fig.6, Fig.S6), where we saw that both Rnd1 and Shirin decreased β-catenin levels and reorganized cell-cell adhesions, though this was seen to a much larger extent in Shirin injected embryos. This was consistent with other data on cell-cell adhesion found in Fig.4H, Fig.5K, Fig.8L, Fig. 9M (new), Fig.S8K (new), and Fig.10EH. We have also simplified figure 8 (doublets) to make it easier to interpret and improved the corresponding text in the results. In short, adhesiveness of cells can be determined by the difference in tension at the cell cortex versus that at the contact, a larger difference between the two values, a stronger adhesion. We show that Shirin very efficiently decreases both cortical tension and contact tension in a way that negatively impacts adhesion. However, Rnd1, while capable of lowering cortical tension, has a comparatively much larger impact on contact tension, which positively impacts adhesion.

Additional points

Fig 1G: The authors argue that the ectodermal cells do not migrate, while the data on 2D show a range of migration speed up to 1 um/min, indicating moderate-speed behavior. The authors should phrase these findings with greater precision. Is this baseline level of epithelial movement in vitro consistent with the relative immobility in vivo?

We clarify in the text (paragraph 3 page 3) that this low level of ‘migration’ is in fact due to the cells ‘wobbling’ back and forth as they bleb moving the cell body back and forth while they are in fact still immobilized on FN and do not migrate.

Fig. 1E: The confocal images indicate that vinculin intensity is rather similar between the ectoderm and mesoderm, but the peaks may differ. These differences should be reflected in their image analysis.

If we understand correctly, the reviewer refers to the possibility that the same total amount of “clustered vinculin” would be differentially distributed, i.e. one condition would have smaller but denser clusters. We had early on considered this possibility, which we could discard:

The quantification presented in Fig.1E is a ratio of the signal present in FAs to the total signal present at the ventral surface on the cell.

To measure this ratio, we obviously had to segment “structures” where vinculin intensity was higher than the basal ventral signal, and measure the surface occupied by these structures. We found that in both ectoderm and mesoderm, and in all conditions, this surface varied exactly in the same way as the equivalent proportion of clustered vinculin. In other words, all the vinculin-enriched structures have on average the same vinculin “concentration”. We therefore consider that the ratio of “total vinculin in FAs” to “total vinculin” is the simplest and most faithful parameter to represent vinculin distribution.

Note that if vinculin concentration would have varied between structures, the interpretation of this difference would have been in any case problematic: Which of two structures with the same total amount of vinculin would be more “adhesive”? A small structure with more concentrated vinculin, or a large structure with comparatively less concentrated vinculin?

All functional studies and analysis after ROCK inhibitor treatment lack control treatments with solvent (presumably DMSO). DMSO may affect cell viability and function, therefore appropriate control data should be provided.

DMSO vehicle controls were in fact performed in all cases where we used inhibitors, this is now mentioned in the materials and methods. There was no effect on the cells.

Fig 2. ROCK inhibition increases the migration speed of ectoderm cells, however, not to the extent of migration speed of the mesoderm. Can the authors explain this incomplete functional transition?

We now acknowledge this in the text (paragraph 1 page 5). The likely explanation is that the Rock inhibitors have a global action on Rock and myosin. As noted by the reviewer in his 3^rd^ point, migration requires a proper balance of contractility, which may not be achieved by generic Rock inhibition. Note also that there could also simply be a difference just based on the size of the ectoderm and mesoderm cells (ectoderm cells are significantly smaller).

Fig 3C-D: The cells show surprisingly little cortical actin. Are they healthy? Viability analysis should be performed to exclude toxic effects after morpholino knockdown.

The signals are membrane-YFP in green and vinculin in Red, not actin. These are not culture cell lines, classical “viability” tests are not relevant here. After years of working with these early embryos, we can safely spot “unhealthy” cells. The cells analysed here adhere to FN substrate within minutes, which is in our experience an unambiguous criterion for a healthy cell. Unhealthy cells also typically fail to express fluorescent markers such as those used here. But the key criterion is presented in Fig.S3: Spreading and migration of KD cells are perfectly rescued by short treatment with the Y inhibitor.

The role of Rnd1 and Shirin in vivo is currently in supplementary figure S3, but could be presented as main data.

These data have been now moved to a new main Figure 3.

Fig 4A-C: The cells' appearance seems to differ from other datasets. Are the dot-like structures indeed focal adhesion or rather vacuoles? Toxic effects?

These dots are due to the autofluorescence of yolk platelets, which occurs in both green and red channels, and is more or less visible depending on the focal plane and the level of expression of the fluorescent proteins of interest. They were marked in figure 1 by “y”. We have now also marked them in other figures where they are prominent (Fig.4B,5A’B’C’,7A’’’,B’’’).

Fig 6: The dot-like cad-tom structures are not explained or mentioned in text. The co-localization of Rnd1 and Shirin with vinculin should be quantified over a larger number of cells.

These dots are again yolk platelets, now marked in the figure. We have performed an extensive co-localization analysis of Shirin and Vinculin, graphs have been added, in what is now Fig.7. The numbers validate what was already obvious visually, i.e. strong colocalization of the mutant form of Shirin, and partial colocalization of the wild type form. Quantitative colocalization analysis for Rnd1 does not make much sense: Co-localization is based on thresholding the image to delimit “structures”, i.e. accumulation or heterogeneities in signal. This is uninformative for Rnd1, which is homogenously distributed signal along the ventral (the pixelated signal is noise), thus there is nothing to extract. We mention in the legend that this homogenous distribution was observed in ALL cells.

In the ectoderm ShirinMO experiment, a higher number of single cells was released, compared to Rnd1MO expression, indicating an effect on cell-cell junctions. By immunofluorescence (Fig. 6), particularly Rnd1 locates at cell-cell contacts. How are these data connected to the expected roles of Rnd1 and Shirin at cell-cell junctions?

We assume that the reviewer refers to the ectopic expression in the ectoderm (Shirin mRNA, not MO). Yes, indeed the trend for more detachment upon Shirin expression is consistent with Rnd1 locating at c-c contacts. And indeed, localization of an inhibitor of Rho-myosin at cell contacts is predicted to decrease local contractility (contact tension) and thus “favour” adhesion. This observation is now commented, as it is consistent with all the other results, including contact tension and adhesiveness from cell doublets, viscosity data, the new IF data on β-catenin and pMLC, as well as explant migration data. We have indeed now a whole body of coherent data confirming the distinct activities of these two regulators to drive more “cohesive” or “dispersive” migration shown in Fig.9I-M and Fig.S8F-K.

Movie 5 and 6: It is unclear what each subpanel shows, as the labels for experimental conditions are missing.

We have corrected the labels on the movies so now they are present on all frames, not just the first.

Figures: Several y axis labels are lacking, e.g. Fig. 1E,H, Fig. 2E,F,H

We have added relevant y axis labels.

Statistical analysis is unclear. In several figures it is unclear which experimental condition was compared to which control, e.g. in Fig 3 and Fig S4. Lines should be included in the figure, and definitions for p values are missing in all legends. The figure legend should further include information about analysis and number of independent experiments.

We now reverted to the standard convention of statistical comparisons with black stars positioned on top of brackets works for the simplest graphs (e.g. Fig.1). However, for more complex graphs, this representation is heavy and difficult to read, especially when multiple conditions must all be compared to control ectoderm or control mesoderm. This would require to superimpose multiple lines/brackets. We find that the possibility to use colours is an opportunity to clarify the presentation of these data. We thus use a consistent colour code for the columns/boxes and for the corresponding statistical comparisons (blue star for comparison with the blue column of control ectoderm, red star for comparison with the red column of control mesoderm). We do use black asterisks and brackets for additional comparisons between specific conditions. We have made sure to clearly explain the colour code in the legend of figure 1, and then use it consistently for all figures. We also explain a definition for p-values in the legend for figure 1 that is used consistently across all figures. For every figure, either the legend or the figure itself shows the number of independent experiments as well as individual measurements, typically represented in brackets as (# of measurements/# of individual experiments).
