# Supplementary material for: Ectoderm to mesoderm transition by down-regulation of actomyosin contractility
Source: PLoS Biol. 2021 Jan 6;19(1):e3001060. doi: 10.1371/journal.pbio.3001060 (PMC7815211; doi:10.1371/journal.pbio.3001060)
Supplement: Attachment — Submitted filename: Response de reviewers 2.docx [file pbio.3001060.s033.docx]

**Response de reviewers**

Rev. 2:

The authors have addressed my comments satisfactorily and in my view the article is much improved by the additional experiments. Before publication, I have one remaining major issue that must be addressed. This is the relationship giving TAB/CtB (relationship (4)).

If I take the expressions (1) and (2) provided in the appendix and try to work out the relationships, I find something different from relationship (4).

Expression (2) can be rewritten to give CtA as a function of TAB and CtB:

(see attachment)

Which is different from (4) in the appendix. My guess is that (4) is in fact TAB/CtA. There is also an alpha that appears in their relationship, which I guess is a typo.

The authors should recheck their appendix and reevaluate everything that derives from (4). It would also be much clearer if they used some equation editing software – like Latex or the word equation editor. Relationship (6) is very difficult to read and could be simplified by noting that (TAB/CtA)/(TAB/CtB) is in fact CtB/CtA.

We thank the reviewer his/her impressively thorough re-reading of the manuscript and for having spotted this gross mistake in the appendix, for which we apologize. The actual equation that was used for our calculations is the exact same one that was obtained by the reviewer. Thus, all our calculated contact tension/ cortical tension values were correct.

We now provide the right equation in the appendix (which by the way should be equation (3), not (4), numbering now corrected). We have also simplified relationship (6) (now (5)) and rewritten all equations using the word editor.

Rev. 3:

The authors introduce the term "ectoderm to mesoderm transition", or "mesoderm transition" to label the fluidization of an epithelium and transition to collective movement. While appropriate for their system, this label may be misleading when epithelia become activated, initiate collective movement, but do not reach mesoderm position or function, such as during wound healing of glandular sprouting. Thus, the authors should reconsider their terminology, or justify the introduction of new terms by detailing the lack of already existing nomenclature. Additional discussion on peculiarities of the in vitro/in vivo comparison aid the general appreciation of methodological choices and limitations.

Lastly, new triangulation data (suggested by referee 1) add depth to the morphological transition analysis. In summary, I applaud to this comprehensive revision.

The term “ectoderm to mesoderm transition” is used to distinguish this transition to a highly motile non-polarized tissue from EMT, which involves additional transformations, in particular loss of polarity. We have further clarified this the introduction (2^nd^ paragraph, 7^th^ line).

We had already discussed that equating this process to tissue “fluidization” would be inaccurate (viscosity is only marginally increased). We have now included in the discussion a short paragraph mentioning the possibility that downregulation of myosin-dependent contractility may be a general principle to generate motility, which may also be part of the more complex EMT-like transition observed in other types of gastrulation.
